# Computational studies of 2-(4-oxo-3-phenylthiazolidin-2-ylidene)malononitrile

**DOI:** 10.1186/s13065-019-0542-6

**Published:** 2019-02-18

**Authors:** Yahia N. Mabkhot, Salim S. Al-Showiman, A. Barakat, S. M. Soliman, Nabila A. Kheder, Mohammed M. Alharbi, Abdulrahman Asayari, Abdullatif Bin Muhsinah, Asad Ullah, Syed Lal Badshah

**Affiliations:** 1 Department of Pharmaceutical Chemistry, College of Pharmacy, King Khalid University, Abha, 61441 Saudi Arabia; 2 Department of Chemistry, College of Science, King Saud University, P. O. Box 2455, Riyadh, 11451 Saudi Arabia; 3 Department of Chemistry, Faculty of Science, Alexandria University, P.O Box 426, Ibrahimia Alexandria, 21321 Egypt; 4 Department of Chemistry, Rabigh College of Science and Art, King Abdulaziz University, Jeddah, 21589 Saudi Arabia; 5 Department of Chemistry, Faculty of Science, Cairo University, Giza, 12613 Egypt; 6 Department of Pharmacognosy, College of Pharmacy, King Khalid University, Abha, 61441 Saudi Arabia; 7 Department of Chemistry, Islamia College University Peshawar, Peshawar, 25120 KPK Pakistan

**Keywords:** Thiazole, DFT/B3LYP calculations, Molecular docking, NBO calculations

## Abstract

The molecular structure of the 2-(4-oxo-3-phenylthiazolidin-2-ylidene) malononitrile (**3**) is calculated using DFT B3LYP/6-311G(d, p) method. The calculated geometric parameters are in good agreement with the experimental data. The NBO calculations were performed to predict the natural atomic charges at the different atomic sites and study the different intramolecular charge transfer (ICT) interactions occurring in the studied system. The BD(2)C17–C19 → BD*(2)C14–C15, LP(2)O2 → BD*(1)N5–C9 and LP(1)N5 → BD*(2)C10–C11 ICT interactions causing stabilization of the system by 23.30, 30.63 and 52.48 kcal/mol, respectively. The two intense electronic transition bands observed experimentally at 249 nm and 296 nm are predicted using the TD-DFT calculations at 237.9 nm (f = 0.1618) and 276.4 nm (f = 0.3408), respectively. These electronic transitions are due to H-3 → L (94%) and H → L (95%) excitations, respectively.

**Electronic supplementary material:**

The online version of this article (10.1186/s13065-019-0542-6) contains supplementary material, which is available to authorized users.

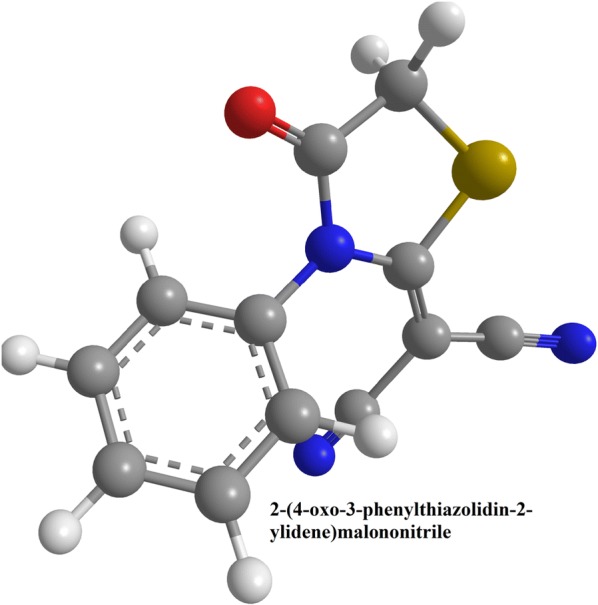

## Introduction

Thiazoles are an important class of heterocyclic compounds that possess the sulphur and nitrogen beside carbon atoms in its five member ring [1]. They are part of a number of pharmaceutical drugs that have analgesic (meloxicam) [2], antihistamine (nizatidine) [3], antibacterial (penicillin) [4], antifungal (thiabendazole) [5], antiprotozoal [6], and a number of other biological properties [1]. They are also part of the essential vitamin B1 or thiamine [7]. In the past, several thiazolidine derivatives have been synthesized and their molecular structural properties have been studied both experimentally and theoretically [8]. In this article we have selected a thiazole based derivative that we have synthesized previously and here we performed density functional theory (DFT) based calculation for its molecular structure [9–11]. The current studied will provide more chemical information about our previously synthesized compound that has good biological activities. These current theoretical studies will further assist in the design and syntheses of better bioactive analogues of thiazole in the future. The 2-(4-oxo-3-phenyl-1,3-thiazolidin-2-yl-idene)malononitrile is a thiazole based derivative that possess several biological properties [10]. We calculated both electronic and spectroscopic properties and compared with previous experimental results of its crystal structure [9, 10, 12, 13]. From the density functional theory (DFT) based calculations we predicted its non-linear optical properties etc. that are discussed below [10]. The DFT will provide information about geometry of the molecule, different orbitals calculations like frontier molecular orbitals will provide information about the π electronic system and intramolecular charge transfer, natural bond orbitals will provide information about different bond interactions and their energies. Similarly, molecular electrostatic potential shows the reactive and non-reactive centers in the molecules while the ultra-violet visible (UV–Vis) spectrum and infra-red (IR) spectrum will also be obtained from these calculations.

## Computational methods

### Quantum chemical calculations

The DFT calculations for the thiazole derivative was performed with the hybrid function of B3LYP and basis set of 6-311G(d, p) [14–16] present in Gaussian 03 software [10, 17]. The coordinates file of the X-ray crystal structure of the thiazole derivative (compound **3**) was downloaded from the online repository [10, 18]. The molecular geometry of the compound **3** was optimized through the energy minimization process without any geometrical parameters constraints [10]. The Gauss View 4.1 [19] and Chemcraft [20] softwares were used for drawing the refined structure of the compound **3** [10]. The energy minima of the optimized geometry of the selected molecule was established as there were no imaginary frequency modes. The electronic, orbital bonding and spectral properties of the selected molecule were also computed through DFT method [21–23]. The natural bond orbital and molecular electrostatic potential analyses for the thiazole derivative was carried out using the B3LYP/6-311G (d, P) level [14–16]. The NBO analyses provides the intramolecular interaction inside the thiazole derivative, stabilization energies and bond interactions. The second order perturbation energy calculation provided the donor and acceptor energies [21–23]. The molecular electrostatic potential analysis produced the most reactive sites in a molecule and thus it is easy to predict the electrophilic and nucleophilic attack sites.

### Docking studies

The molecular docking was executed on the molecular operating environment (MOE) 2014.09 software [24–26]. The atomic coordinates of the human B-lactate dehydrogenase in complex with oxidized form of nicotinamide adenine dinucleotide and 4-hydroxy-1,2,5-oxadiazole-3-carboxylic acid having PDB ID Number 1T2F was downloaded from protein data bank website [27]. The structure of the protein and the selected ligand was optimized, and energy minimization was performed. The binding pockets in the protein receptor were determined with site finder module of MOE [24, 25]. The efficiency of the docking program was gauged by re-docking the original ligand into the established receptor active site for the determination of root mean square deviation (RMSD) [26]. After that, the malononitrile compound 3 was docked with the receptor protein and the conformer with best docking score and free energy was selected [26, 28].

## Results and discussion

### Chemistry

**Fig. 1:**
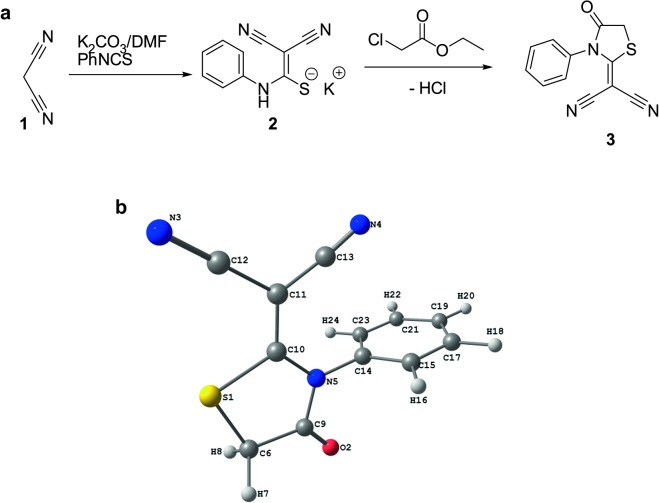
**a** Synthesis of thiazole derivative **3** [9]. **b** Optimized chemical structure of the malononitrile analogue

Malononitrile was stirred with phenyl isothiocyanate in K_2_CO_3_ in dimethylfluoride to afford an intermediary anionic compound 2, which on reacting with ethyl chloroacetate forming the targeted molecule 3 [9] (Fig. 1a).

### Optimization of the compound geometry

The optimized molecular structure bond lengths and their angles were calculated through the hybrid function of B3LYP with 6-311G(d, p) basis set as tabulated in Table 1; and compared with the experimentally determined optimized molecular structure from the literature [9, 29]. This compound possess C_1_ point group and its optimized structural information were compared with the crystallographic information file (CIF) [9, 10]. All the predicted geometric parameters agree with the experimental results. The bond distances of the compound **3** are a little overestimated except the C11‒C12 bond which is shorter by 0.009 Å than the experimental one [30]. The main deviations in the values of calculated from the experimental bond length and angle are 0.041 Å (S1–C) and 1.3° (O2–C9–C6), respectively [30]. The predicted values of C‒C‒C bond angle of the phenyl ring of compound **3** are in the range of 119.1‒120.3° while the experimental values are 117.9‒120.8° [10, 31]. The calculated dihedral angles of the phenyl and thiazole rings of this molecule are close to 0° showing a planar structure [10] (Fig. 1b).

**Table 1:** Comparison of different geometric parameters of malononitrile analogue [32]

Parameter	Calc.	Exp	Parameter	Calc.	Exp
R(1-6)	1.824	1.783	A(2-9-5)	124.0	123.6
R(1-10)	1.769	1.739	A(2-9-6)	124.9	126.2
R(2-9)	1.199	1.191	A(3-12-11)	179.1	179.4
R(3-12)	1.157	1.132	A(4-13-11)	175.2	175.1
R(4-13)	1.156	1.137	A(9-5-10)	116.8	116.7
R(5-9)	1.411	1.399	A(9-5-14)	118.8	119.1
R(5-10)	1.377	1.357	A(5-9-6)	111.1	110.2
R(5-14)	1.447	1.444	A(10-5-14)	124.4	124.2
R(6-9)	1.518	1.501	A(5-10-11)	127.9	126.9
R(10-11)	1.376	1.367	A(5-14-15)	119.3	118.9
R(11-12)	1.425	1.434	A(5-14-23)	119.3	119.0
R(11-13)	1.423	1.426	A(10-1112)	118.1	118.1
R(14-15)	1.391	1.370	A(10-1113)	126.0	126.8
R(14-23)	1.391	1.368	A(12-1113)	115.9	115.0
R(15-17)	1.391	1.384	A(15-1423)	121.4	122.1
R(17-19)	1.393	1.366	A(14-1517)	119.1	117.9
R(19-21)	1.393	1.361	A(14-2321)	119.1	119.0
R(21-23)	1.391	1.365	A(14-2324)	120.0	120.4
A(6-1-10)	92.1	92.1	A(15-1719)	120.1	120.1
A(1-6-9)	107.8	108.5	A(17-1921)	120.3	120.8
A(1-10-5)	112.1	112.3	A(19-2123)	120.1	120.1
A(1-10-11)	119.9	120.8			

### Natural atomic charge on the molecule

The charge distribution over a molecule has pivotal role in quantum chemistry. The atomic charges are related to the electronic density, charge distribution and dipole moment of a compound. The natural atomic charges (NAC) computed through DFT at the different atomic positions are tabulated in Table 2. The studied molecule has oxygen, nitrogen and sulfur-heteroatoms. The O and N-atoms are the most electronegative atomic spots in the malononitrile analogue [33]. In contrast, the S-atom is electropositive. The calculated natural atomic charge for the two N-sites of the nitrile groups (N3 and N4) are approximately equivalent [33]. While the NAC at the thiazole nitrogen atom is more negative than the N-atoms of the nitrile group. In the present compound **3**, all the H-atoms are electropositive whereas the aliphatic protons (H7 and H8) are more positively charged than the aromatic ones [33]. The NAC on the aliphatic and aromatic protons are 0.2437 and 0.2080–0.2146, respectively. Most of the aromatic C-atoms are electronegative except C14 as this carbon bonded to the high electronegative N5-atom [34]. The most electropositive C-atom in the molecule is the carbonyl carbon [32].

**Table 2:** The natural atomic charges theoretically measured for the malononitrile derivative

Atom	NAC	Atom	NAC
S1	0.3360	C13	0.2843
O2	− 0.5350	C14	0.1276
N3	− 0.3001	C15	− 0.1827
N4	− 0.3016	H16	0.2146
N5	− 0.4890	C17	− 0.1768
C6	− 0.5793	H18	0.2096
H7	0.2437	C19	− 0.1724
H8	0.2437	H20	0.2080
C9	0.7180	C21	− 0.1768
C10	0.1830	H22	0.2096
C11	− 0.3820	C23	− 0.1827
C12	0.2855	H24	0.2146

### Molecular electrostatic potential

**Fig. 2:**
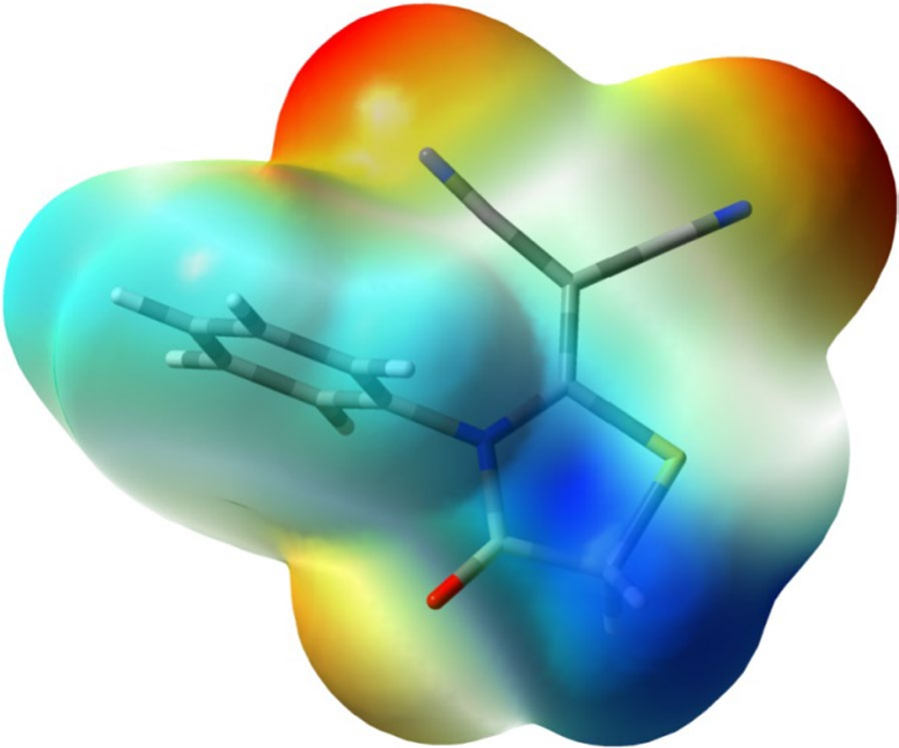
The molecular electrostatic potentials map predicted for the malononitrile analogue

The distribution of charge and its related properties of compounds can be obtained through the 3D electrostatic potential maps. The electrostatic potential map was produced by overlapping the Van der Waal’s radii of each atoms present in the compound **3** so that it reveals the charged surface and thus one can visualize the morphological properties of the molecule [10, 35, 36]. Through these maps, we can forecast the reactive spots for electrophilic as well as the nucleophilic attack during the chemical reactions [10, 37, 38]. The malononitrile derivative electrostatic potential map was predicted through the same DFT hybrid function and basis set as other parameters were measured and is presented in Fig. 2. The charged surface map in Fig. 2 showed that the negative regions (red) contain the N3 and N4 atoms of the nitrile group, showing that these N-sites are the hot spots for electrophilic attack. While the blue regions in Fig. 2 represent the positive regions that contain the area of H7, H8 and C6-atoms of the compound **3** and are the hot spot of nucleophilic attacks. These results gave information about how compound **3** interact with receptor active sites.

### Nonlinear optical properties

The nonlinear optical materials are important for photonic communications due to its use light for data transmission and thus are an actively used in industry nowadays [39–41]. Many organic based compounds are used in photonic communication instruments due to their superior polarizability (α_0_) and lower energy gap (ΔE) between their highest occupied and lowest unoccupied molecular orbitals [40–42]. Here the α_0_ and ΔE values of the thiazole based malononitrile derivative are 162.89 Bohr^3^ and 4.6905 eV, while the polarizability value is approximately six times that of urea. Based on our calculations it has lower ΔE than urea. Thus, this thiazole based compound has superior nonlinear optical qualities than the reference molecules [33, 43, 44].

### Frontier molecular orbitals (FMOs) of the malononitrile analogue

**Fig. 3:**
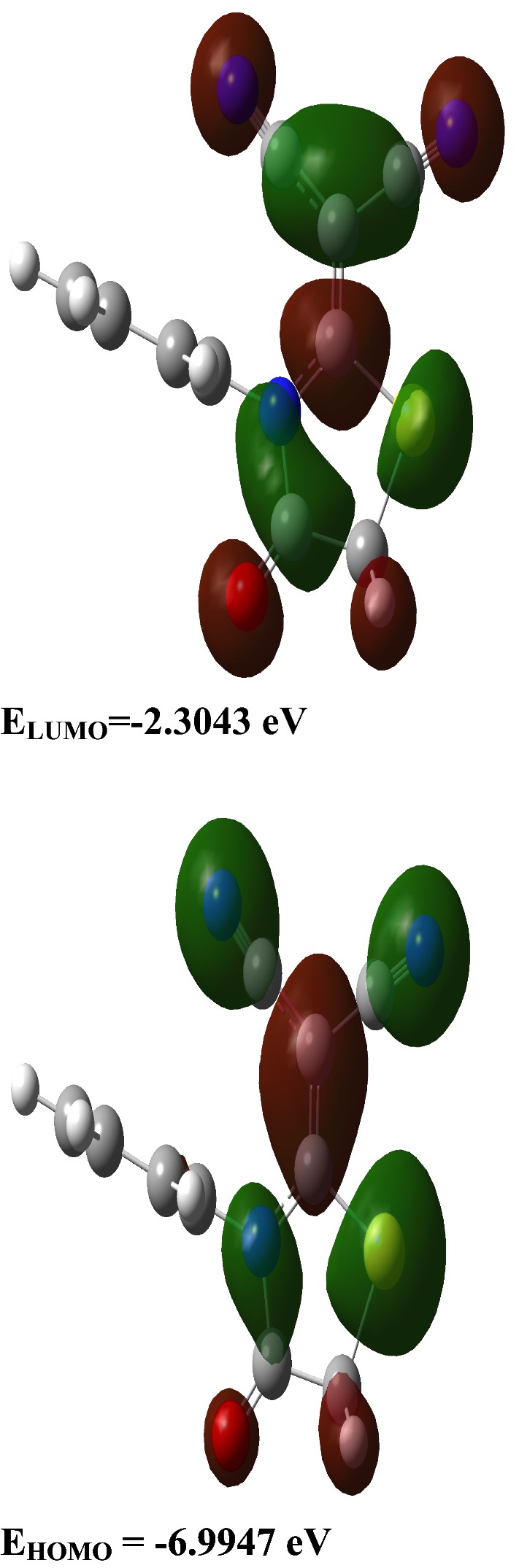
Electronic density surface plots at ground state for the FMOs of thiazole based malononitrile analogue. The molecular orbitals level are delocalized over the five member thiazole ring and the attached cyanate groups. Green represent negative values of the orbital overlap, dark red represents positive values of the orbitals overlap [50]

The electronic densities of FMOs are helpful in predicting the reactive positions and different reaction types for a π-electron systems containing molecules [33, 43]. Further, the energies of the two types of orbitals (E_HOMO_ and E_LUMO_) and their ΔE of a molecule showed its inherent chemical reactivity and intramolecular charge transfer (ICT) capacities [32, 45–48]. The ΔE for the FMOs of the thiazole based compound **3** was calculated through the hybrid function of B3LYP/6‒311G (d, p) and its FMOs picture is presented in Fig. 3 [32]. It was observed that the molecular orbitals level are delocalized over the five member ring of the compound and the C10–C11–C–N π-electronic systems. The E_HOMO_ is − 6.9947 eV while E_LUMO_ is − 2.3043 eV. The orbitals ΔE signifies a lower energy electronic transition with a value of 4.6905 eV for the thiazole based compound under study. This ICT of electron transition happens due to π–π* excitations. The 40 spin allowed singlet–singlet electronic transitions predicted are tabulated in Additional file 1: Table S1 and the electronic spectrum is presented in Additional file 1: Figure S4 [49]. Experimentally there are two intense electronic transition bands that are observed at 249 nm and 296 nm. On the basis of DFT calculations, these electronic spectral bands were observed at 237.9 nm (f = 0.1618) and 276.4 nm (f = 0.3408) on the spectrum and these can be assigned to the excitation from H-3 → L (94%) and H → L (95%) respectively.

### Natural bond orbital (NBO) analysis

The stabilization energies E^(2)^ for the relevant intra-molecular charge transfer contacts were calculated through the NBO method (Table 3) [51, 52]. The different types of interactions between filled and empty orbitals in a complex molecule can be used to measure the intra-molecular electronic density delocalization. A larger stabilization energy showed high rate of electronic exchange between donor and acceptor NBOs, i.e. higher the amount of conjugation inside the molecule [53]. The second-order perturbation theory is used to describe the energetics of such interactions [10, 54, 55]. The intramolecular charge transfer exchange as a result of the orbital overlap between π → π*, n → σ* and n → π* orbitals helps in stabilization of the molecular system up to 23.30, 30.63 and 52.48 kcal/mol respectively, which are due to BD(2)C17–C19 → BD*(2)C14–C15, LP(2)O2 → BD*(1)N5–C9 and LP(1)N5 → BD*(2)C10–C11 ICT interactions, respectively [10]. These predicted results showed that there is strong electronic density spread from LP(1)N5 to the nearby C10–C11π*-NBO. Further, there is a π → π* electron delocalization between the nitrile group π-system to the nearby π*-NBO of the C10–C11 bond in this thiazole based compound.

**Table 3:** The stabilization energies E^(2)^ in (kcal/mol) of the important charge transfer interactions between the donor and acceptor in the malononitrile analogue

Donor NBO (i)	Acceptor NBO (j)	E^(2)^ kcal/mol
BD(1)N3–C12	BD*(1)C11–C12	8.09
BD(3)N3–C12	BD*(2)C10–C11	7.66
BD(1)N4–C13	BD*(1)C11–C13	8.49
BD(3)N4–C13	BD*(2)C10–C11	8.50
BD(1)C10–C11	BD*(1)C11–C12	5.29
BD(1)C10–C11	BD*(1)C11–C13	5.93
BD(2)C10–C11	BD*(3)N3–C12	20.67
BD(2)C10–C11	BD*(3)N4–C13	19.65
BD(2)C10–C11	BD*(2)C10–C11	6.94
BD(1)C11–C12	BD*(1)N3–C12	8.23
BD(1)C11–C12	BD*(1)N5–C10	6.32
BD(1)C11–C12	BD*(1)C10–C11	5.01
BD(1)C11–C13	BD*(1)N4–C13	8.66
BD(1)C11–C13	BD*(1)C10–C11	6.46
BD(2)C14–C15	BD*(2)C17–C19	17.82
BD(2)C14–C15	BD*(2)C21–C23	20.47
BD(1)C15–C17	BD*(1)N5–C14	5.05
BD(2)C17–C19	BD*(2)C14–C15	23.30
BD(2)C17–C19	BD*(2)C21–C23	20.11
BD(1)C21–C23	BD*(1)N5–C14	5.05
BD(2)C21–C23	BD*(2)C14–C15	21.81
BD(2)C21–C23	BD*(2)C17–C19	20.50
LP(2)S1	BD*(2)C10–C11	26.02
LP(2)O2	BD*(1)N5–C9	30.63
LP(2)O2	BD*(1)C6–C9	22.18
LP(1)N3	BD*(1)C11–C12	12.47
LP(1)N4	BD*(1)C11–C13	12.59
LP(1)N5	BD*(2)O2–C9	44.57
LP(1)N5	BD*(2)C10–C11	52.48

### Vibrational spectrum analyses

The IR vibrational spectrum of the malononitrile analogue were computed through the same hybrid function and basis set as described previously and the vibrational modes were assigned through visual inspection through the GaussView software [10, 17, 56]. The comparison between theoretically computed and the experimental vibrational band frequencies of the compound **3** are tabulated in Additional file 1: Table S2 and the IR spectrum is shown in Fig. 5 [10]. The Additional file 1: Table S2 showed that there is close resemblance between the two IR vibrational frequencies [10, 57].

#### Aromatic C–H bending vibrations

The thiazole ring of the malononitrile derivative posseses the carbon–hydrogen stretching vibrations in 3100–3000 cm^−1^ region [10, 58]. In this study, the IR band recognized for the C–H stretching vibrations at 3042 cm^−1^ is present at 3097–3070 cm^−1^ [10, 59]. The in-plane and out-of-plane ring C–H bending vibrations bands are predicted in the region 1400–1000 and 1000–600 cm^−1^ respectively [10, 60, 61]. The DFT analyses also showed that the in-plane bending modes are at 1475, 1438, 1305, 1158, 1148, 1128, 1067 and 1013 cm^−1^ (exp. 1461, 1291, 1157 and 1024 cm^−1^) [10]. Few of the in-plane C–H bending vibrational modes mixed with other bands [62]. On other hand, the out-of-plane bending modes for C–H are present at 980–904, 817, 739 and 684 cm^−1^ (exp. 991, 911, 791 and 698 cm^−1^) [10]. Thus our theoretical calculated C–H vibrational frequencies agree very well with experimental data.

#### Aliphatic C–H vibrations of the thiazole ring

The compound under investigation has one methylene group at the thiazole ring. It will therefore exhibits two aliphatic C–H stretching vibration at lower frequencies in the spectrum than those of the aromatic C–H ring vibrations [63–65]. Both the symmetric and asymmetric stretching vibrations of the CH_2_ group are present at 3027 (exp. 2997 cm^−1^) and 2979 cm^−1^ (exp. 2944 cm^−1^), in the computed vibrational spectrum [62, 66, 67]. The theoretically computed CH_2_ scissoring, wagging, twisting and rocking vibrations are present at 1410 cm^−1^ (exp. 1384 cm^−1^), 1285 cm^−1^ (exp. 1234 cm^−1^), 1109 cm^−1^ and 888 cm^−1^ (exp. 886 cm^−1^), respectively [10, 64].

#### C≡N vibrations

Generally the nitrile stretching vibration is present at 2250 ± 10 cm^−1^ in saturated nitriles or in olefinic nitriles where no conjugation exists between the nitrile and the olefinic group [68, 69]. While in conjugated nitrile, the band moves to lower frequency of 2225 ± 7 cm^−1^ [69, 70]. Here in this molecule, there are two nitrile groups attached to the C=C so the symmetric and asymmetric υ_C≡N_ modes are predicted at 2251 and 2241 cm^−1^ [71]. In agreement with literature, the υ_C≡N_ mode observed experimentally at 2215 cm^−1^. In the IR spectrum of this compound **3**, the symmetric and asymmetric δ_C–C≡N_ modes were predicted at 602 and 462 cm^−1^ (exp. 468 cm^−1^), respectively. Furthermore, the nitrile group out-of-plane torsion mode has a predicted at 454 cm^−1^ (exp. 453 cm^−1^) [10, 64, 65, 72].

#### C=O, C=C and C–S vibrations

In compounds that have aromaticity, the C=C stretching vibrations is mostly present at 1600–1500 cm^−1^ [63, 73]. In the current system, these stretching vibrations are predicted at 1587–1438 cm^−1^ while experimentally observed at 1597–1461 cm^−1^ [10]. We also found that the aromatic rings breathing modes are at 987 cm^−1^ (exp. 999 cm^−1^) [10]. We noted, the υ_(C10=C11)_ stretching mode at 1520 cm^−1^ while experimentally it is observed at 1527 cm^−1^ [10]. The malononitrile analogue showed intense carbonyl vibration observed experimentally at 1745 cm^−1^ (calc. 1769 cm^−1^). The thiazole ring C–S stretching vibrational frequency mode is calculated at 758 cm^−1^ while it is experimentally noted at 757 cm^−1^ [69].

### Molecular docking

**Fig. 4:**
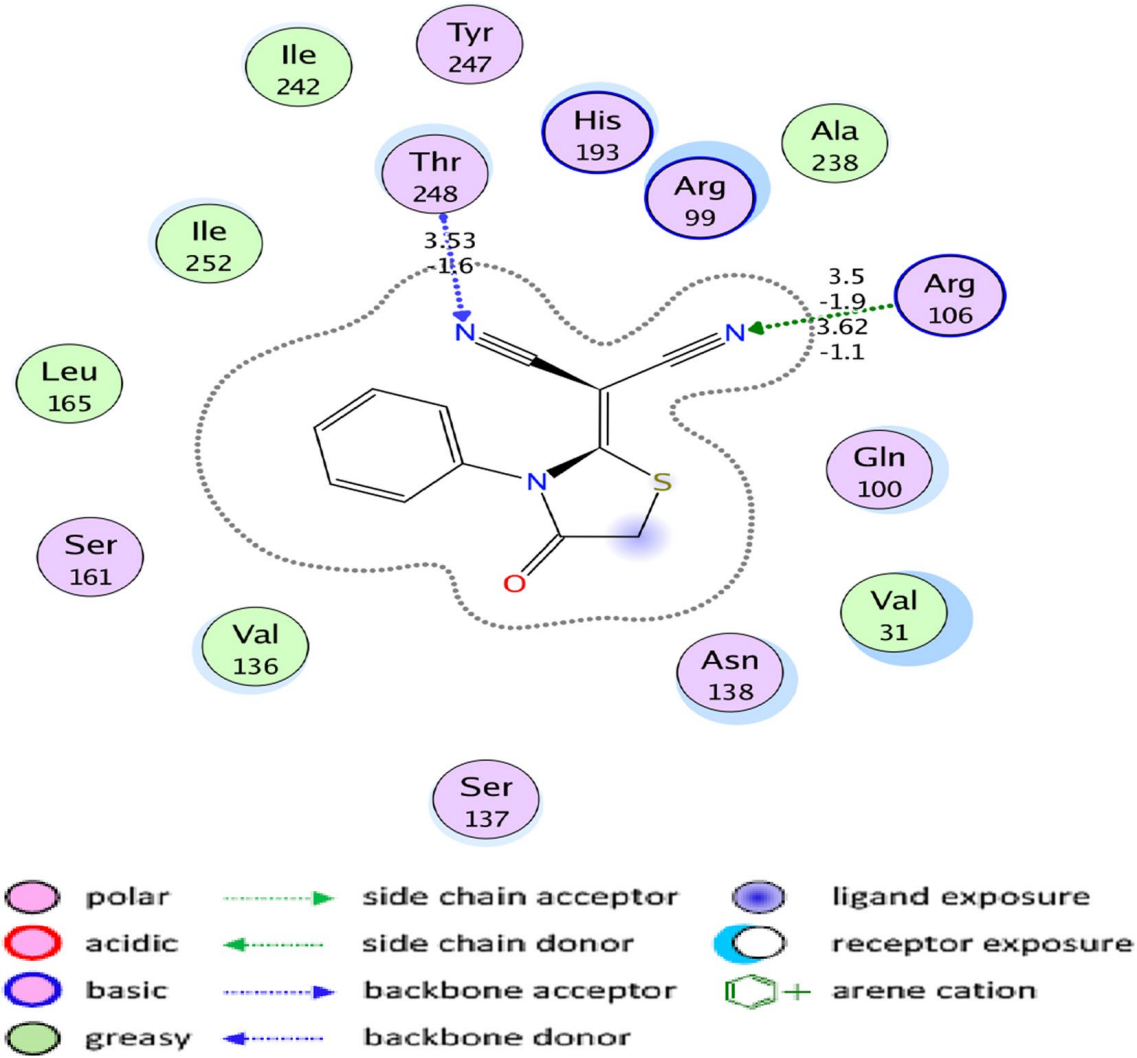
2D representation showing the hydrogen bond interactions of the interesting compound and the targeting enzyme

**Fig. 5:**
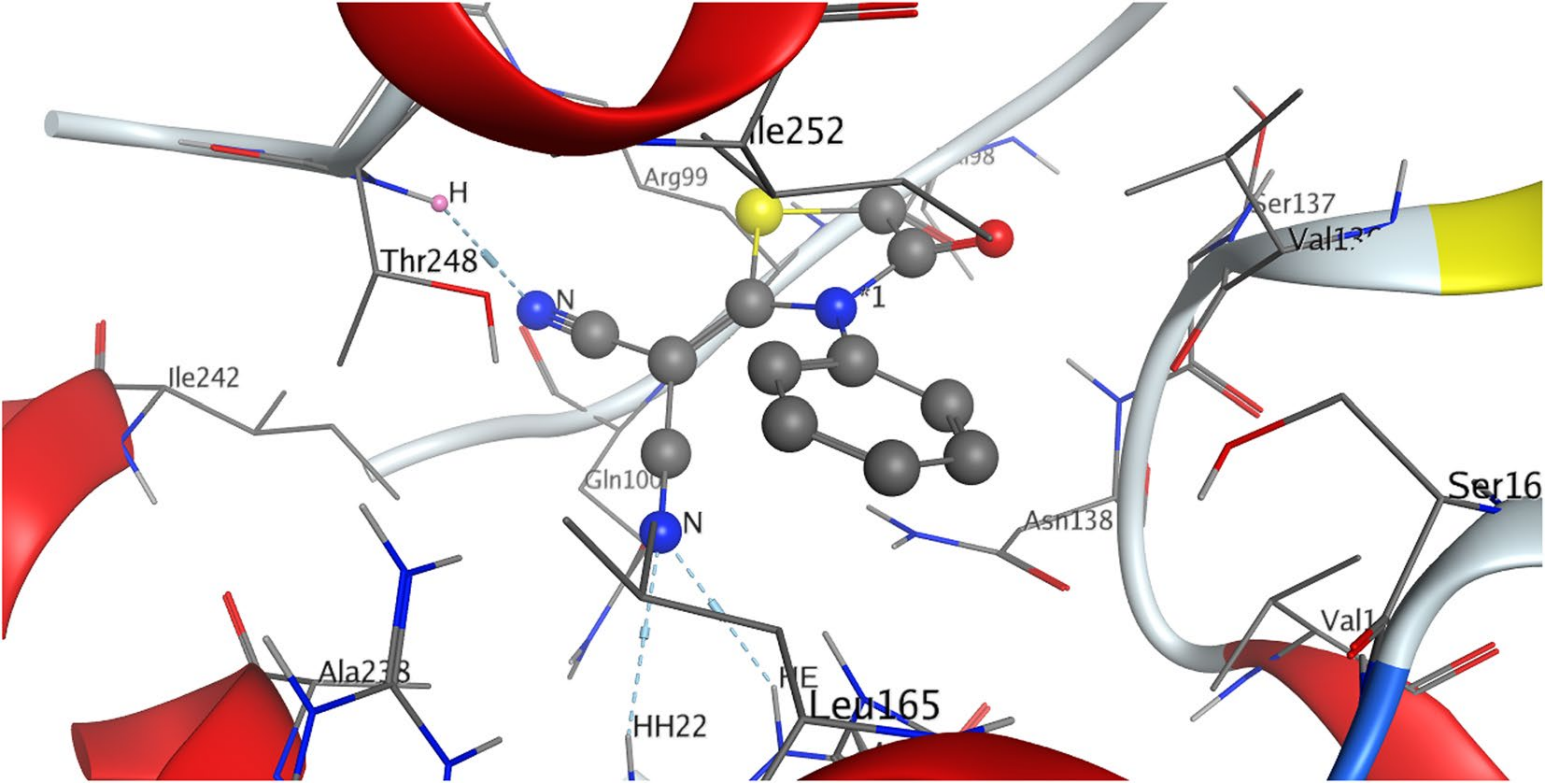
3D representation of the selected compound pose fitted inside the targeted enzyme

Lactate dehydrogenase (LDH) has an active role in the metabolism of lactate during normal physiological process [74, 75]. The high levels of lactate are associated in different ways to several types of human cancers, as cancerous cells have increased metabolism [75]. The increase level of lactate ion may directly contributes to tumor growth and progression [75]. Molecular docking was conducted to find out the interaction of 2-(4-oxo-3-phenylthiazolidin-2-ylidene) malononitrile with the lactate dehydrogenase enzyme. The malononitrile derivative has good affinity for the LDH enzyme showing a total free energy of − 4.6 kcal/mol on its interaction. It is clearly the cyano moiety that is the highly active group in the malononitrile by making two hydrogen acceptor interactions with **Arg 106** with − 3.0 kcal/mol and one hydrogen acceptor interaction with **Thr 248** with − 1.6 kcal/mol (Figs. 4, 5).

### Pharmacophore studies

**Fig. 6:**
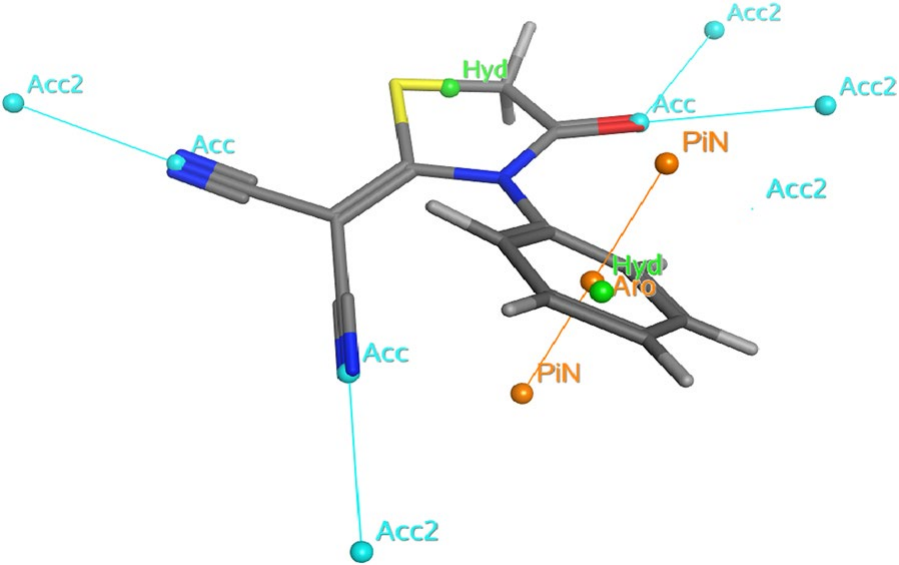
Pharmacophore annotations of the malononitrile analogue

The pharmacophore analyses was done with the MOE software package (version 2014.010) using the default settings. The different conformers production for the under test ligand was carried out using the conformational analysis algorithm, present in the MOE software package [76]. Pharmacophore modeling tools determine the different chemical properties and spatial arrangement in three dimensions that are essential for interaction between ligand and its receptor and thus for the drug action. Pharmacophore models can be generated from the structural data of protein–ligand complexes as well as from ligands when no receptor information is available and also from the receptor structure when no ligands are available. The generated models are usually used for virtual screening of online libraries of compounds that are the potentially active molecules. Also, the pharmacophoric feature may represent a specific property and is not necessarily related to a particular chemical structure; but different chemical groups may share the same property and possesses the same feature (Fig. 6 and Table 4) [77, 78].

**Table 4:** Pharmacophore annotations of compound **3**

Pharmacophore	Annotation
Number of hydrogen bond acceptor atoms	3
Number of acidic atoms	0
Number of basic atoms	0
Number of hydrogen bond donor atoms	0
Number of hydrogen bond donor and hydrogen bond acceptor atoms	3
Number of hydrophobic atoms	10
Approximation to the sum of VDW surface areas (Å2) of pure hydrogen bond acceptors	49.052
Approximation to the sum of VDW surface areas of acidic atoms (Å2)	0
Approximation to the sum of VDW surface areas of basic atoms (Å2)	0
Approximation to the sum of VDW surface areas of pure hydrogen bond donors (Å2)	0
vsa_hyd	134.17
Approximation to the sum of VDW surface areas (Å2) of atoms typed as “other”	53.55
Approximation to the sum of VDW surface areas (Å2) of polar atoms (atoms that are both hydrogen bond donors and acceptors), such as –OH	49.052

VDW: van der Waals; vsa_hyd: Approximation to the sum of VDW surface areas of hydrophobic atoms (Å2)

## Conclusions

The chemical structure of the 2-(4-oxo-3-phenylthiazolidin-2-ylidene) malononitrile was optimized through the hybrid B3LYP method and 6-311G (d, p) basis set. The predicted geometrical parameters of the malononitrile derivative agree well with the previous experimental results. From this study it was observed that this malononitrile compound has superior non-linear optical properties than urea. Thus, it can be used in photonic communication instruments.

## Additional file

**Additional file 1: Table S1.** The calculated electronic transition bands of the malononitrile compound. **Table S2.** Comparison of the predicted and experimental frequency modes of the malononitrile analogue. **Figure S1.** Comparison of the predicted (upper) and experimental (lower) absorbance spectra of the thiazole based malononitrile analogue. **Figure S2.** The experimental (lower one) and calculated (upper one) IR spectra of the studied compound.

## Abbreviations

DFT: density functional theory
B3LYP: Becke, 3-parameter, Lee–Yang–Parr
CIF: crystallographic information file
MOE: molecular operating environment
PDB: protein data bank
RMSD: root mean square deviation
K_2_CO_3_: potassium carbonate
HOMO: highest occupied molecular orbitals
LUMO: lowest unoccupied molecular orbitals
NBO: natural bond orbitals
NAC: natural atomic charge
FMO: frontier molecular orbital
eV: electron volt
MEP: molecular electrostatic potential
ICT: intramolecular charge transfer
