# Supplementary material for: Computational studies of 2-(4-oxo-3-phenylthiazolidin-2-ylidene)malononitrile
Source: BMC Chem. 2019 Feb 18;13(1):25. doi: 10.1186/s13065-019-0542-6 (PMC6661733; doi:10.1186/s13065-019-0542-6)
Supplement: Supplementary file 1 — Additional file 1: Table S1. The calculated electronic transition bands of the malononitrile compound. Table S2. Comparison of the predicted and experimental frequency modes of the malononitrile analogue. Figure S1. Comparison of the predicted (upper) and experimental (lower) absorbance spectra of the thiazole based malononitrile analogue. Figure S2. The experimental (lower one) and calculated (upper one) IR spectra of the studied compound. [file 13065_2019_542_MOESM1_ESM.docx]

**Additional Information**

**Table S1: The calculated electronic transition bands** **of the malononitrile compound.**

| **λ_max_ (nm)** | **f** | **Major contributions** |
| --- | --- | --- |
| **276.2** | **0.3408** | **H→L (95%)** |
| 267.0 | 0.0000 | H→L+1 (90%) |
| 265.8 | 0.0003 | H-2→L (99%) |
| 263.3 | 0.0019 | H-1→L (100%) |
| 247.2 | 0.0004 | H-4→L (89%) |
| **237.9** | **0.1618** | **H-3→L (94%)** |
| 233.2 | 0.0003 | H→L+3 (78%) |
| 231.6 | 0.0071 | H→L+2 (99%) |
| 231.5 | 0.0038 | H-2→L+2 (35%), H-1→L+1 (53%) |
| 219.8 | 0.0177 | H→L+4 (96%) |
| 216.5 | 0.0001 | H-3→L+1 (29%), H→L+5 (57%) |
| **211.9** | **0.0784** | **H-2→L+1 (84%), H-1→L+2 (12%)** |
| 210.7 | 0.0000 | H-3→L+1 (53%), H→L+5 (38%) |
| 207.0 | 0.0002 | H-5→L (92%) |
| 201.9 | 0.0013 | H-1→L+3 (10%), H→L+6 (71%) |
| 201.1 | 0.0116 | H-2→L+2 (11%), H-1→L+1 (21%), H-1→L+3 (47%), H→L+6 (18%) |
| 199.0 | 0.0000 | H-2→L+4 (91%) |
| 197.3 | 0.0090 | H-1→L+2 (11%), H-1→L+4 (81%) |
| 195.3 | 0.0108 | H-4→L+1 (11%), H-2→L+3 (48%), H-1→L+2 (23%), H-1→L+4 (15%) |
| 195.1 | 0.0002 | H-7→L (87%) |
| 194.2 | 0.0332 | H-4→L+1 (83%) |
| 191.9 | 0.0001 | H-3→L+3 (85%) |
| 191.1 | 0.0003 | H-4→L+4 (79%) |
| 190.3 | 0.0114 | H-6→L (86%) |
| 189.9 | 0.0038 | H-3→L+2 (97%) |
| **181.7** | **0.2611** | **H-3→L+4 (63%), H-1→L+2 (13%)** |
| 181.1 | 0.0042 | H-4→L+2 (95%) |
| **179.6** | **0.1305** | **H-5→L+1 (17%), H-2→L+5 (14%), H→L+8 (37%)** |
| 178.6 | 0.0798 | H-2→L+2 (12%), H-1→L+3 (14%), H-1→L+5 (43%) |
| 177.2 | 0.0628 | H-8→L (30%), H-4→L+3 (40%), H-3→L+4 (10%) |
| 176.8 | 0.0158 | H-9→L (36%), H→L+7 (30%) |
| 176.6 | 0.0082 | H-9→L (60%), H→L+7 (18%) |
| 174.8 | 0.0265 | H-5→L+1 (16%), H-2→L+5 (44%), H→L+8 (15%) |
| **173.8** | **0.1681** | **H-2→L+2 (20%), H-1→L+3 (13%), H-1→L+5 (36%), H-1→L+6 (10%)** |
| 173.4 | 0.0738 | H-2→L+3 (11%), H-2→L+5 (28%), H-1→L+2 (13%) |
| 172.3 | 0.0025 | H-10→L (70%), H-1→L+6 (25%) |
| 172.2 | 0.0296 | H-8→L (15%), H-4→L+3 (13%), H-2→L+6 (60%) |
| 171.9 | 0.0806 | H-10→L (23%), H-1→L+6 (55%) |
| 171.6 | 0.0272 | H-8→L (35%), H-4→L+3 (17%), H-2→L+6 (29%) |
| 170.8 | 0.0000 | H→L+9 (81%) |

**Table S2:** Comparison of the predicted and experimental frequency modes of the malononitrile analogue.

| **Assignment** | **Calculated** | **Experimental** |
| --- | --- | --- |
| υ_(CH, aromatic)_ | 3097-3070 | 3042 |
| υ_(CHasym, CH2)_ | 3027 | 2997 |
| υ_(CHsym, CH2)_ | 2979 | 2944 |
| υ_(C≡N, sym)_ | 2255 | 2215 |
| υ_(C≡N, asym)_ | 2241 |  |
| υ_(C=O)_ | 1769 | 1745 |
| υ_C=C_ | 1587, 1582, 1520^a^, 1475,1438 | 1597, 1527 ^a^, 1461 |
| δ_(CH, sciss.)_ | 1410 | 1384 |
| δ_(CH, wag.)_ | 1285 | 1234 |
| δ_CH aromatic in plane_ | 1475, 1438, 1305, 1158, 1148, 1128, 1067, 1013 | 1461, 1291, 1157, 1024 |
| δ_(CH, twist.)_ | 1109 | - |
| Ring breathing | 987 | 999 |
| δ_CH aromatic out-of-plane_ | 980-904, 817, 739, 684 | 991, 911, 791, 698 |
| δ_(CH, rocking.)_ | 888 | 886 |
| υ_(C-S)_ | 758 | 757 |

**υ: streching δ: bending ^a^**υ_(C10=C11)_

**
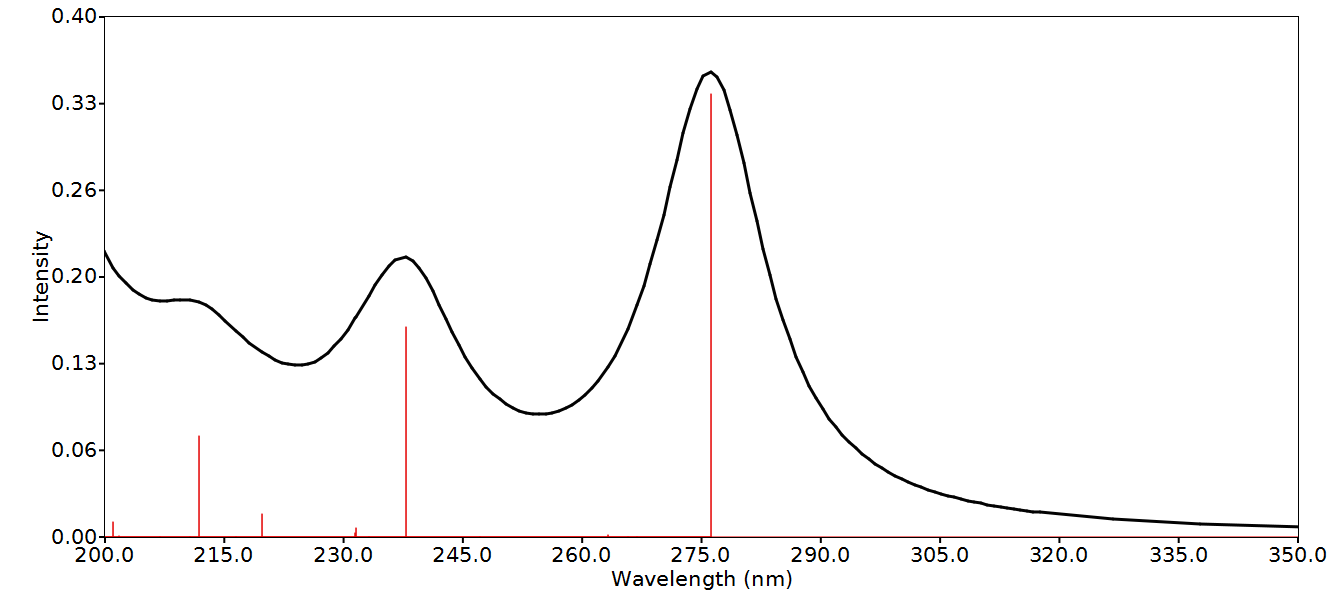
**

**
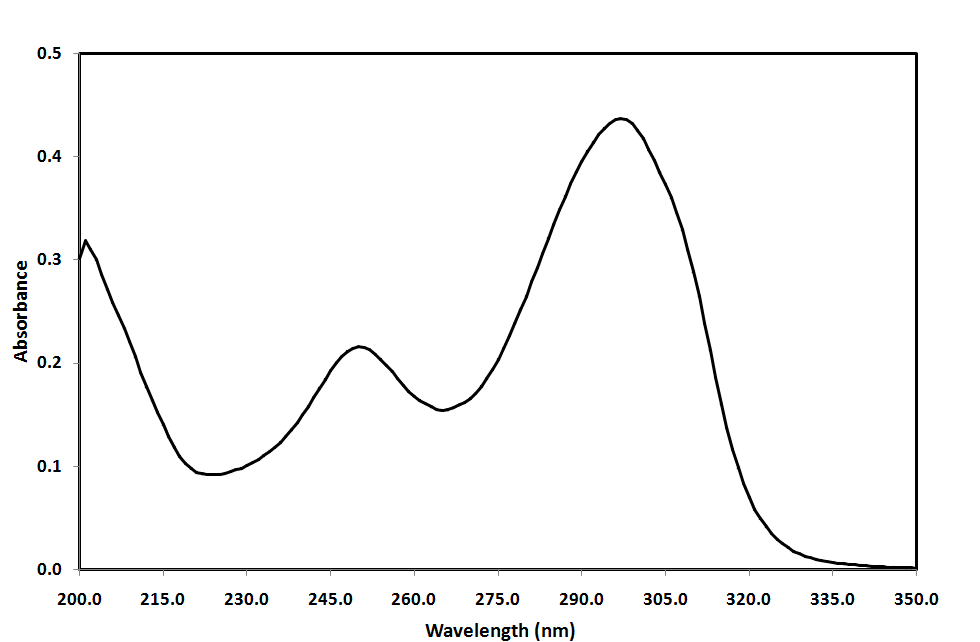
**

**Figure S1:** Comparison of the predicted (upper) and experimental (lower) absorbance spectra of the thiazole based malononitrile analogue.

**
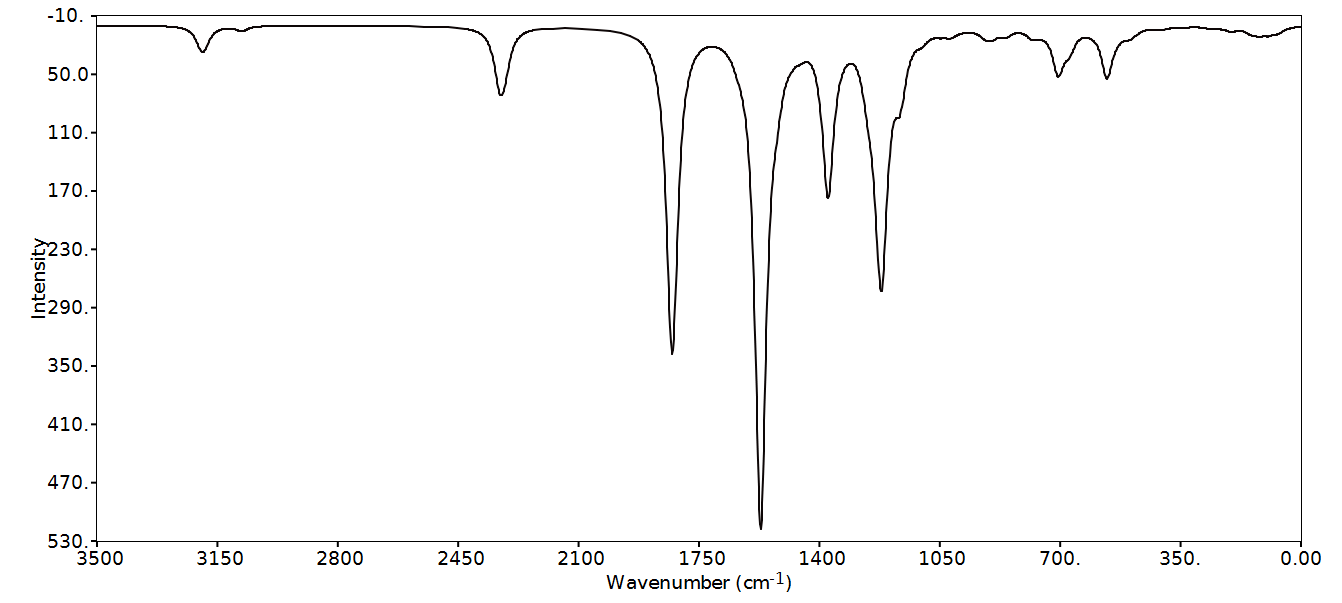
**

**Figure S2:** The experimental (lower one) and calculated (upper one) IR spectra of the studied compound.
